# Assessment of the Mechanical Parameters of Resin Composites with the Addition of Various Types of Fibres

**DOI:** 10.3390/ma13061378

**Published:** 2020-03-18

**Authors:** Bernardeta Dębska, Lech Lichołai, Guilherme Jorge Brigolini Silva, Marina Altoé Caetano

**Affiliations:** 1 Department of Building Engineering, Rzeszow University of Technology, ul. Poznańska 2, 35-959 Rzeszów, Poland; Lech.Licholai@prz.edu.pl; 2 Departamento de Engenharia Civil, Universidade Federal de Ouro Preto, Campus Morro do Cruzeiro, Ouro Preto CEP 35.400.000, Brazil; guilhermebrigolini@ufop.edu.br (G.J.B.S.); altoemarina@gmail.com (M.A.C.)

**Keywords:** epoxy mortars, fibres, mechanical properties, Cracked Straight Through Brazilian Disc test, microstructural analysis

## Abstract

The article describes tests of epoxy mortars after the addition of fibres. The fibres were a substitute for sand in the amount of 0, 1, 2, 3, 4 and 5% by volume, respectively. Three types of mortar were obtained, containing polypropylene, glass and carbon fibres, respectively. Statistical analyses (ANOVA) were carried out to assess the impact of fibre content on the mechanical properties of mortars. Brittle fracture toughness was also tested using the Cracked Straight Through Brazilian Disc method. The addition of each type of fibre improved the assessed parameters. Based on the obtained research results, and also due to availability and price, the most advantageous seems to be the production of composites containing the addition of polypropylene fibres.

## 1. Introduction

Resin concretes and mortars are composites that do not contain cement. The binder in this type of materials is a synthetic resin, most often polyester, epoxy, and less often acrylic. Bonding and hardening occur as a result of mixing this type of resin with an appropriate amount of a properly selected hardener [1,2,3]. The aggregate used to make resin composites must be durable and clean as well as dry. Most often it is quartz sand [4], but partial sand replacement with alternative aggregates, such as perlite [5], expanded clay [6], and waste rubber [7,8] gives good effects. Resin concretes are characterized by very high compressive strength (over 100 MPa) and bending strength, as well as very good chemical resistance. Their characteristic feature is also the short time needed to achieve operational efficiency and their good adhesion to various building materials [4,9]. Their high strength-to-weight ratio, good damping properties and ability to form complex shapes make them ideal for prefabrication [10,11]. These properties mean that resin composites can be used as anti-corrosive shields used in adverse environmental conditions, in the construction of bridge, road, hydrotechnical, marine and urban buildings. Resin sewer and telecommunications wells and their components can also be obtained. Resin industrial floors and elements of bridge drainage systems (cornices, curbs, drains) and prefabricated elements for linear drainage systems (channels, gutters, wells) are popular. Resin concretes are used for the production of resin marble, as well as for joining structural elements and emergency repairs of buildings. Due to the high cost of polymers, resin mortars are only used in special cases. However, their practical properties make them appear on the market more and more often. Unfortunately, the price is not the only disadvantage of these materials. They are also characterized by limited heat and fire resistance and relatively high curing shrinkage. Due to the presence of the resin matrix, these materials exhibit quasi-fragile behaviour [12,13,14,15]. However, this feature can be improved, among others by incorporating fibres into the composite mix. Resin mortars with the addition of fibres were obtained by, among others, [16,17,18,19,20,21]. In most cases, it has been confirmed that fibres addition is important, as it allows significant improvement of the mechanical properties of resin composites, and at the same time makes them more resistant to cracking. The mechanics of resin mortar cracking were studied in detail by Reis et al. [16,22,23,24,25,26]. The properties of such composites largely depend on the characteristics of their constituent materials and the interactions between them [27]. What are important, among others, are fibre-matrix adhesion and fibre length, internal/external diameter and structure of the fibres, fibre treatment and dispersion in the matrix [28]. The mechanics of cracking in polymer mortar is strongly dependent on the fibre orientation [15]. In this work, a study was undertaken on the selection of the optimal amount of various types of fibres added to mortars, which will guarantee the obtaining of the best physical and mechanical properties of the obtained composites.

## 2. Materials and Methods 

### 2.1. Materials

#### 2.1.1. Binder

The binder in the obtained mortars was Epidian 5 epoxy resin, CIECH Sarzyna S.A., Nowa Sarzyna, Poland. The resin content was 36% vol. The hardener was triethylenetetramine (hardener Z-1), added in an amount of 10% by weight based on the weight of the resin. Selected resin and hardener parameters are presented in Table 1 and Table 2.

#### 2.1.2. Aggregate

The aggregate was quartz sand, KWARCMIX, Tomaszów Mazowiecki, Poland, with a density of 2.65 g/cm^3^ and a grain size of 0–2 mm, in accordance with the requirements of the standard PN-EN 196: 2016 [30].

#### 2.1.3. Fibres

**Figure 1:**
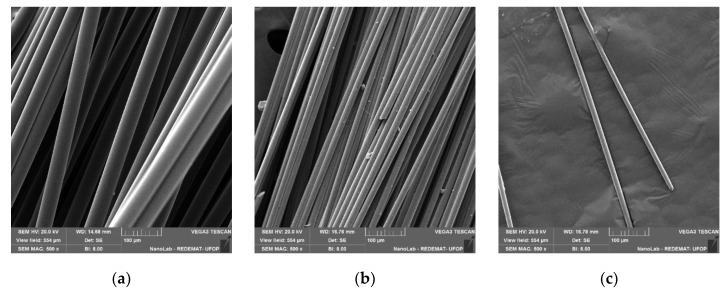
SEM images at 500× magnification of (**a**) polypropylene; (**b**) glass and (**c**) carbon fibres.

**Figure 2:**
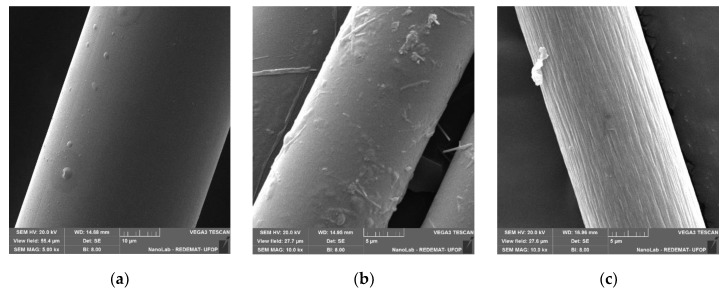
SEM images of (**a**) polypropylene at 5000× magnification; (**b**) glass and (**c**) carbon fibres at 10,000× magnification.

Three types of 12 mm long commercially available fibres, Rozenblat Sp. z o.o., Krosinko, Poland, were used to obtain the mortars: polypropylene (pp), glass (g) and carbon (c). Moreira et al. [16] suggested that the addition of an amount of fibre exceeding 2% wt. no longer strengthens the resin mortar, but in other works a positive effect of fibres was noted at 4%, 8% [1] and even 10% of the additive [14]. In the tests described in this article, the fibres were added in an amount of 0, 1, 2, 3, 4 and 5% by volume of aggregate. Selected fibre properties are summarized in Table 3. Photographs of fibres from a scanning microscope at 500× and 10,000× magnification are shown in Figure 1 and Figure 2, respectively.

### 2.2. Sample Preparation

Appropriate amounts of individual components were weighed. The resin was mixed with the hardener and poured into the bowl of the mixer. Then sand mixed with fibres was added. The whole composition was mixed in a laboratory mixer at a speed of 140 ± 5 rpm, maintaining a fixed mixing time of 3 minutes. Epoxy mortars were cast into standard prismatic moulds (40 × 40 × 160 mm^3^) for flexural and compressive tests and into cylindrical moulds (ø80 mm, h = 38 mm) for testing brittle fracture toughness. The samples were ripened in the laboratory in room environment for 7 days and then tested.

### 2.3. Methods

#### 2.3.1. Bending Flexural Strength Test

The flexural strength when bending composites was tested using a three-point bending test in accordance with PN-EN 196: 2016 [30]. Three samples from each composition were used. The sample was loaded at a speed of 0.25 mm/min. The equipment for testing flexural strength was a universal testing machine (Cometech Testing Machines Co., Taichung, Taiwan) with a maximum load capacity of 50 kN, 

#### 2.3.2. Compressive Strength Test

These were carried out on the halves of samples formed after the flexural strength test (six samples from each series). A hydraulic press (MATEST S.p.A., Arcore, Italy) equipped with test inserts ensuring a constant compression area equal to 1600 mm^2^ was used. The samples were loaded at a speed of 2.4 kN/s.

#### 2.3.3. Brittle Fracture Toughness

**Figure 3:**
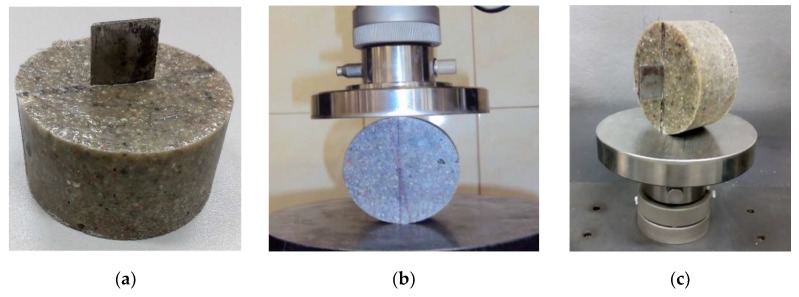
Sample for testing brittle fracture toughness: (**a**) before testing; (**b**) in the testing machine; (**c**) after the test.

This test was conducted based on the method called Cracked Straight Through Brazilian Disc (CSTBD, Cometech Testing Machines Co., Taichung, Taiwan) which was described in detail in articles [16,31]. This is an alternative method to the commonly used one, and its benefit is that it allows reduction of the cost of obtaining samples, because in this case they are much smaller. Based on the work of Moreira et al. [16,31], cylindrical samples with a diameter of 80 mm and a height of 38 mm were made, including control samples (without the addition of fibres) and for all of the types of fibres, the amount of which in the composite was set at 2%. This approach allowed comparison of the obtained test results. A fracture was simulated by inserting thin metal washers 25 mm wide and 2 mm thick in the centre of the cylinder (Figure 3a). 

The stress intensity factors (K_IC_; K_IIC_) were calculated based on the formulas (1)–(4) [31]:

(1) $$K_{I}=\frac{F}{\sqrt{\pi }\cdot a\cdot w}\cdot \sqrt{l}\cdot N_{I}$$

(2) $$K_{II}=\frac{F}{\sqrt{\pi }\cdot a\cdot w}\cdot \sqrt{l}\cdot N_{II}$$

(3) $$N_{I}=\left(1-4\cdot {\left(\sin\left(\theta \right)\right)}^{2}\right)\cdot [T_{1}+T_{2}\cdot {\left(\frac{l}{a}\right)}^{2}\cdot 8\cdot {\left(\sin\left(\theta \right)\right)}^{2}]$$

(4) $$N_{II}=2\cdot \sin\left(2\theta \right)\cdot [S_{1}+S_{2}\cdot {\left(\frac{l}{a}\right)}^{2}\cdot \left(8\cdot {\left(\cos\left(\theta \right)\right)}^{2}-5\right)]$$

The parameters necessary for the calculations were adopted as in [31]: *T_1_* = 1.135551, *T_2_* = 0.533477, *S_1_* = 1.089702, *S_2_* = 0.522272, sample height *w* = 38 mm, sample radius *a* = 40 mm, half the width of the metal plate *l* = 12.5 mm. In formulas (1) and (2), the force causing the destruction of the sample read from the machine was adopted as *F*, and *θ* was the angle of force application in relation to the metal plate.

Samples were tested after 7 days of conditioning under laboratory conditions. A universal testing machine was used with a constant head speed of 0.5 mm/min, fitted with parallel steel plates (Figure 3b).

#### 2.3.4. Scanning Electron Microscopy (SEM)

Scanning Electron Microscopy (SEM), secondary electrons (SE) and back scattering electron (BSE), was performed using a VEGA 3 microscope (TESCAN, Nanolab Laboratory, Federal University of Ouro Preto - UFOP, Ouro Preto, Minas Gerais, Brazil). The samples were obtained from crushed specimens of the mechanical strength test. Polished and unpolished samples were assessed after coating with a thin layer of gold. Realized this experiment to evaluate the dispersion and the behavior of the fibers added to the produced matrices after the load application.

## 3. Results and Discussion

The results of the strength tests performed were subjected to statistical analysis using the Statistica 12 program (StatSoft, Kraków, Poland). Two factors were taken into account, i.e. the type of fibre and the content of fibres. Non-parametric bidirectional ANOVA statistics of the Kruskal-Wallis rank (available in the Nonparametric Statistics tab—Comparison of many independent samples (groups) of the Statistica program) were used to:

- check whether each factor considered independently has a significant impact on the values of flexural and compressive strength,
- determine the main contribution of each factor to global variance.

In all analyses performed, the impact of factors with a significance level less than or equal to 5% (p ≤ 0.05) was considered statistically significant.

### 3.1. Flexural Strength

The results of the flexural strength test for epoxy mortars containing a variable amount and different type of fibres (along with a standard deviation) are summarized in Table 4.

**Figure 4:**
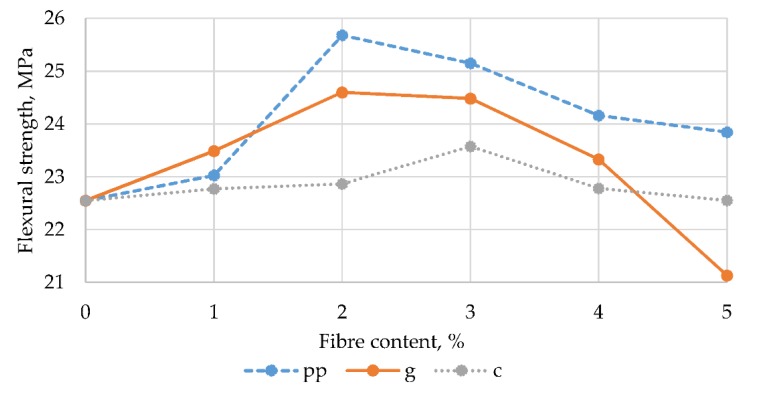
The dependence of flexural strength on the content and type of fibres.

For visual assessment, these results are presented graphically in Figure 4.

Based on the test results obtained, it can be stated that the addition of each of the three types of fibres in an amount of up to 4% causes an increase in flexural strength compared to the control samples. On the other hand, a 5% fibre addition results in a decrease in the determined parameter. Similar behaviour was demonstrated by samples of polyester mortars modified by recycled glass fibre, tested by Ribeiro et al. [1]. These authors noted an increase in strength at 4% and 8% sand substitution with fine fibres, and at 4% modification with thicker fibres. 

In turn, Xie et al. [20] obtained an improvement in flexural strength of epoxy mortars with the addition of 1.6% polypropylene fibres in relation to the weight of the resin, with the most favourable results obtained for a content of 0.8% fibres. It can be seen in Figure 4 that the highest values of flexural strength, at 25.68 MPa, were noted for mortars containing polypropylene fibres added in an amount of 2%. Similar behaviour was demonstrated for mortars with the addition of 2% glass fibres, for which the flexural strength was 24.60 MPa. The flexural strength of mortars modified with carbon fibres was characterized by the lowest variation, increasing from 22.55 MPa for 5% fibre content (the same as for control samples) to 23.58 MPa for samples where the amount of fibres is 3%. These conclusions are supported by the results of the non-parametric analysis of ANOVA Kruskal-Wallis variance, carried out separately for two accepted, changing factors.

#### 3.1.1. Statistical Analysis of Test Results Related to the First Factor (Type of Fibre)

The results of the Kruskal-Wallis ANOVA test for flexural strength and the independent variable–the type of fibre–are given in Table 5. The Kruskal-Wallis test is essentially an analysis of variance carried out on ranks. The test statistics in this test are not significant (p = 0.0672). 

Therefore, it can be concluded that the flexural strength of epoxy mortars modified with three types of fibres does not differ significantly. Analysing the p-values for multiple (two-sided) comparisons presented in Table 6, it can be concluded that only slightly insignificant (p = 0.061) differences in the results of flexural strength of mortars containing carbon and polypropylene fibres can be assumed.

**Figure 5:**
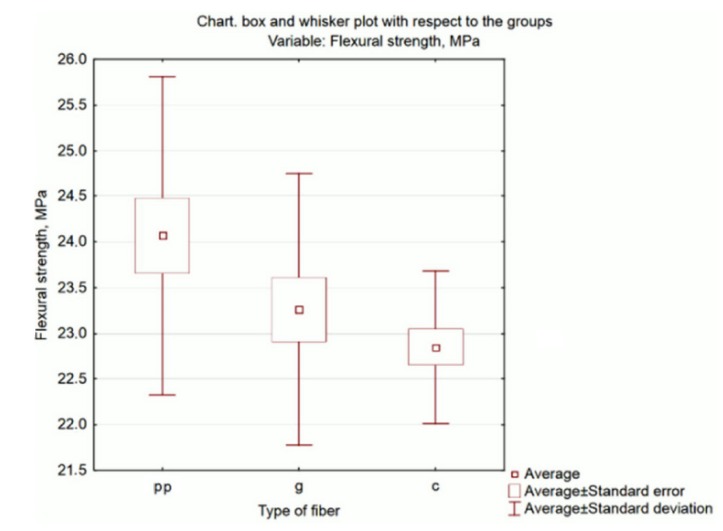
Box and whisker plot of flexural strength related to the type of fibres.

Graphic presentation of the results is possible by generating the box and whisker plot presented in Figure 5.

**Figure 6:**
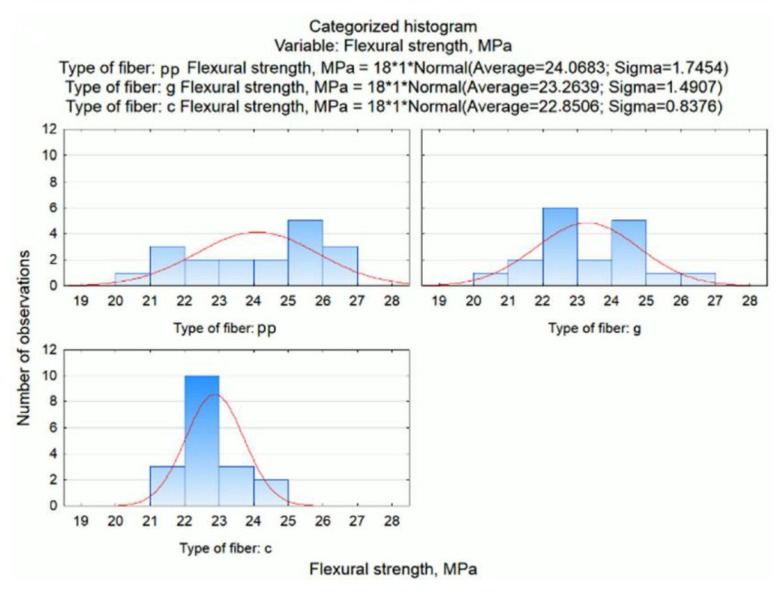
Categorized histograms of flexural strength related to the type of fibres.

Another example of assessing the distribution of a dependent variable (flexural strength) within each of the conditions (type of fibres) is the categorized histogram option. These diagrams are presented in Figure 6. 

It confirms the conclusion that for mortars containing polypropylene fibres, the flexural strength is the highest (i.e. the distribution is shifted to the right towards higher values), lower for mortars with glass fibres and the lowest for mortars modified with carbon fibre.

#### 3.1.2. Statistical Analysis of Test Results Related to the 2nd Factor (Fibre Content) 

The Kruskal-Wallis ANOVA test for flexural strength can also be performed in relation to the second independent variable, which was the percentage of fibres in the composite. The results of this test are given in Table 7. 

In this case, the test statistics are significant (p = 0.0104). Therefore, it can be concluded that the amount of fibres significantly differentiates mortars in terms of flexural strength. Analysing the p-values for multiple (two-sided) comparisons presented in Table 8, it can be stated that the greatest differences in flexural strength occur between control mortars (without fibres) and mortars containing 3% fibres. 

**Figure 7:**
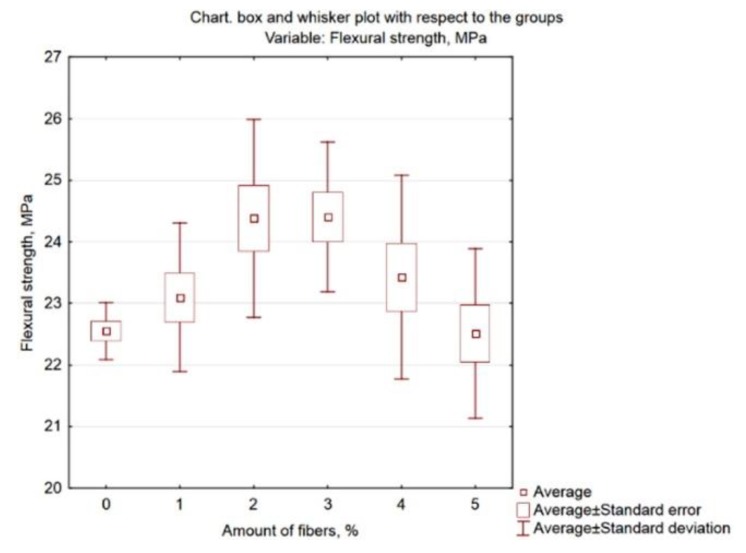
Box and whisker plot of flexural strength related to related to fibre content.

Graphic presentation of these results is possible by generating the Box and whisker plot presented in Figure 7.

**Figure 8:**
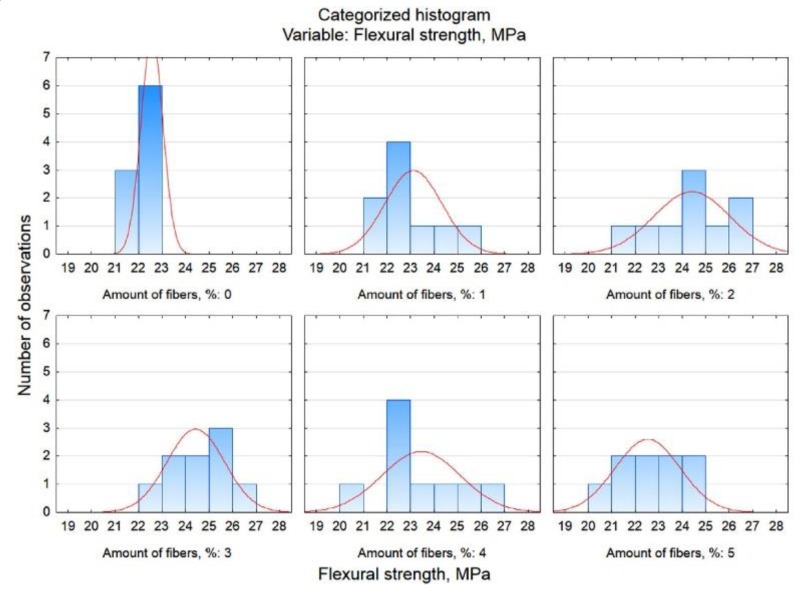
Categorized histograms of flexural strength related to related to fibre content.

The categorized histograms shown in Figure 8 also confirm the analyses performed. For mortars containing 2% and 3% fibres, the flexural strength is the highest–these distributions are shifted to the right towards higher values.

### 3.2. Compressive Strength Test

The results of the compressive strength test for epoxy mortars containing a variable amount and type of fibres together with a standard deviation are summarized in Table 9. 

**Figure 9:**
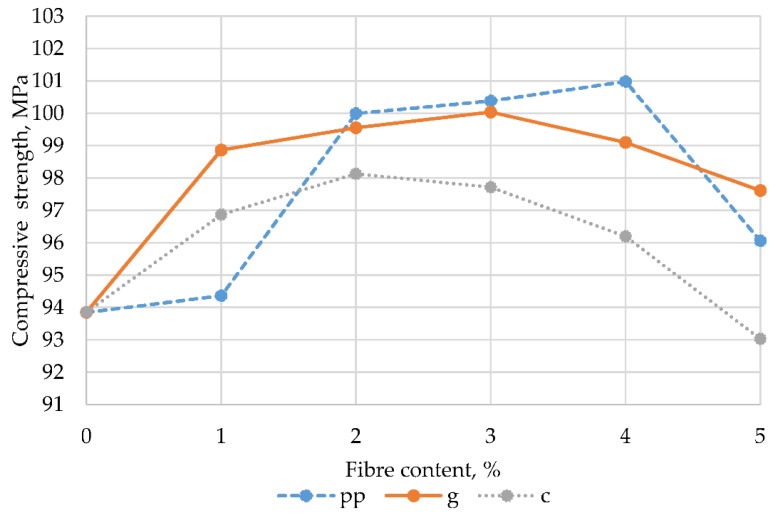
Dependence of compressive strength on the content and type of fibres.

To facilitate interpretation, these results are presented graphically in Figure 9.

The data summarized in Table 9 and presented in Figure 9 confirm that, as in the case of flexural strength, that the addition of fibres allows the obtaining of higher compressive strength values of epoxy mortars in comparison to control samples (without fibres). At low fibre content (1%), the highest increase in compressive strength (compared to the control samples) of 5.35% was noted for glass fibre mortars, slightly lower (3.22%) for carbon fibre mortars and the lowest (0.55%) for samples containing polypropylene fibres. However, a larger proportion (2–4%) of polypropylene fibres resulted in mortars with the most favourable compressive strength values ranging from 100.00 MPa to 100.98 MPa, respectively. Among the mortars containing fibres, the lowest values of compressive strength were characterized by those that were modified with carbon fibres. Comparing these results to the studies described in the literature, whose authors [20] stated that the addition of polypropylene fibres to epoxy mortars gives the best effects at a content of 0.8% and allows the obtaining of compressive strength at a level of 69.4 MPa, it can be stated that they are much lower than those recorded in our research. Lower compressive strength, not exceeding 86 MPa (with 8% addition of recycled fibres), compared to the results presented in this article, was also characteristic of polyester mortars obtained by Ribeiro et al. [1].

The mechanical properties of mortars are significantly influenced by the length, diameter as well as the internal and external structure of the fibres. Larger diameter fibres usually have weaker mechanical properties. The obtaining of the lowest strength parameters for mortars based on carbon fibres may be due to the fact that with more fibres in the process of mixing and forming, the agglomeration of fibres could occur (due to the strong fibre-fibre interaction), which in turn could hinder the ideal homogenization of the mixture. The carbon fibres used for the research were hand cut from rovings. In this process, impurities could be formed on the surface of the fibres. With a larger number of fibres in the composite, the number of micro-pores increases, which also reduces the mechanical parameters.

#### 3.2.1. Statistical Analysis of Test Results Related to the First Factor (Type of Fibre)

Also, in the case of compressive strength, the Kruskal-Wallis ANOVA test was carried out. The results of this test for the independent variable—the type of fibre—are given in Table 10. 

This test showed statistical significance (p = 0.0104). Therefore, it can be concluded that the compressive strength of epoxy mortars modified with the three types of fibres is significantly different from each other. Analysing the p-values for multiple (two-sided) comparisons presented in Table 11, it can be concluded that the differences in the compressive strength results of mortars containing glass and carbon fibres can be considered significant (p = 0.012799). 

**Figure 10:**
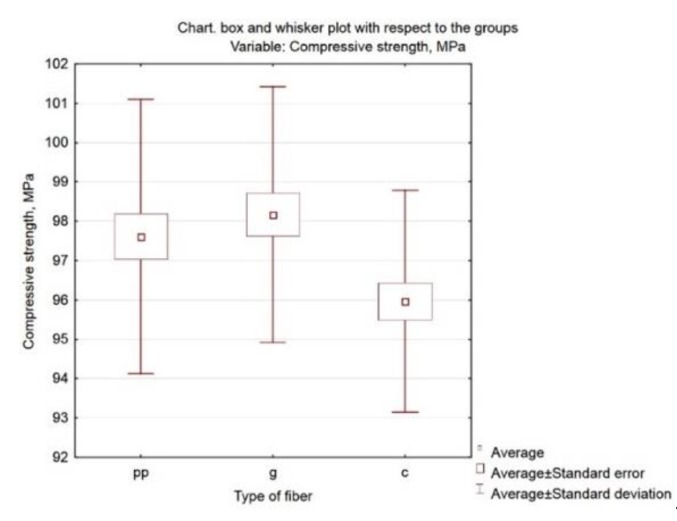
Box and whisker plot of compressive strength related to the type of fibre.

It can also be assumed that the differences in the compressive strength values of mortars with carbon and polypropylene fibres are slightly statistically significant (p = 0.068124). Graphical presentation of the results is possible by generating the box and whisker plot presented in Figure 10. 

**Figure 11:**
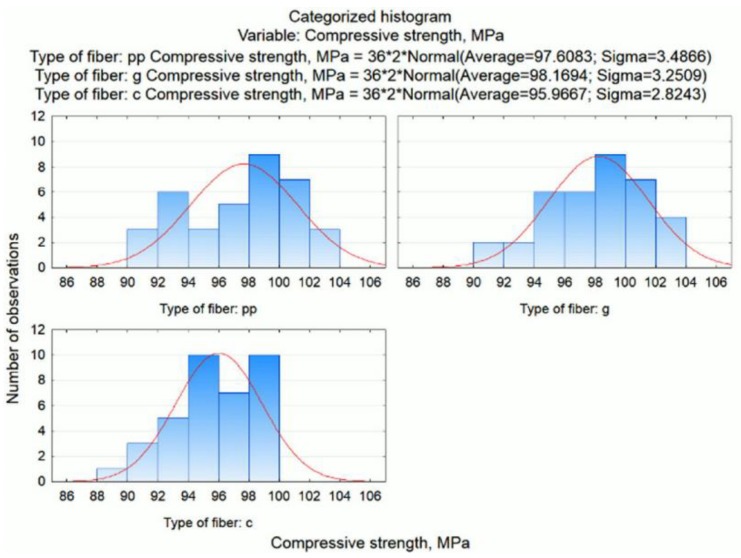
Categorized histograms of compressive strength related to the type of fibre.

The assessment of the distribution of the dependent variable (compressive strength) within each of the conditions (type of fibres) was also carried out based on a categorized histogram. These graphs are shown in Figure 11. 

Here, the conclusion is confirmed that for mortars containing glass fibres, the compressive strength is the highest (i.e. the distribution is shifted to the right towards higher values), slightly lower for mortars with polypropylene fibres and the lowest for mortars modified with carbon fibres.

#### 3.2.2. Statistical Analysis of Test Results Related to the 2nd Factor (Fibre Content)

The Kruskal-Wallis ANOVA test for compressive strength was also carried out in relation to the second independent variable, which was the percentage of fibres in the composite. The results of this test are given in Table 12. 

In this case, the test statistics are highly significant (p = 0.0000). Therefore, it can be concluded that the amount of fibres significantly differentiates mortars in terms of compressive strength. Analysing the p-values for the multiple (two-sided) comparisons presented in Table 13 it can be stated that the greatest differences in compressive strength occur between control mortars (without fibres) and mortars containing 2% and 3% fibres.

**Figure 12:**
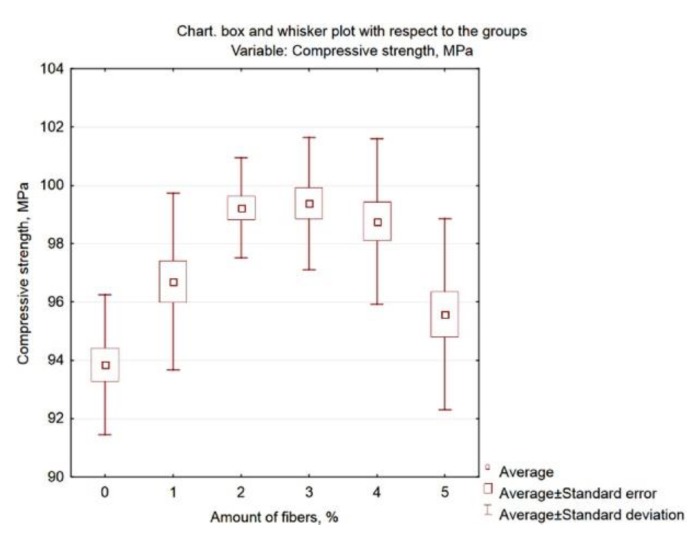
Box and whisker plot of compressive strength related to fibre content.

Graphic presentation of these results is possible by generating the Box and whisker plot presented in Figure 12.

**Figure 13:**
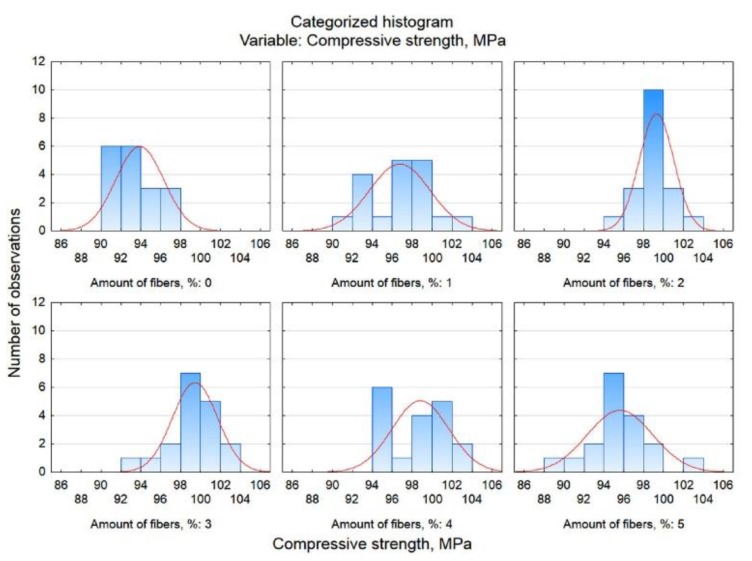
Categorized histograms of compressive strength related to fibre content.

The categorized histograms shown in Figure 13 also confirm the conclusions resulting from the analyses. For mortars containing 2% and 3% fibres, the compressive strength is the highest - these distributions are shifted to the right towards higher values.

### 3.3. Brittle Fracture Toughness

**Figure 14:**
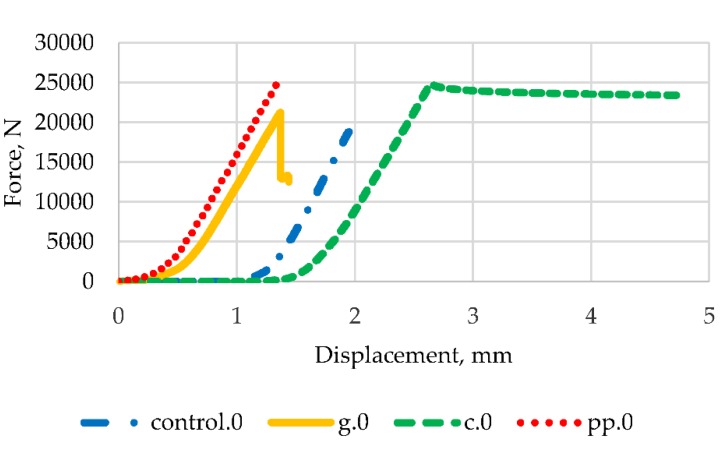
Load vs. displacement curves for 0° angle and all types of mortar.

**Figure 15:**
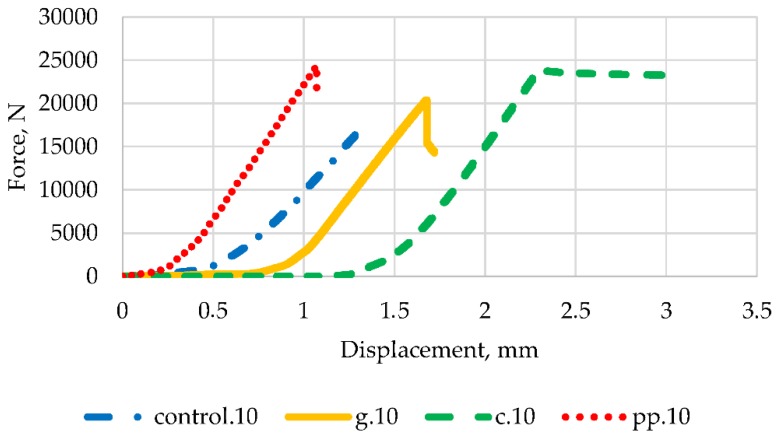
Load vs. displacement curves for 10° angle and all types of mortar.

**Figure 16:**
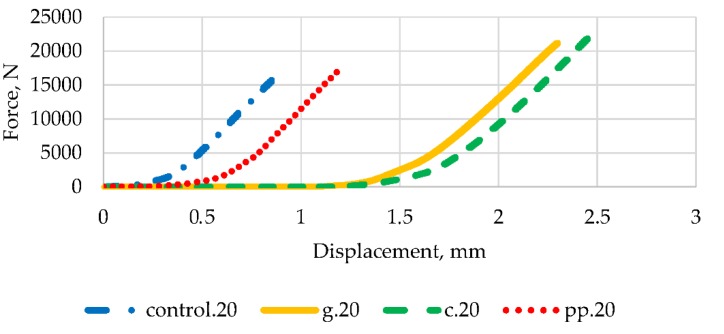
Load vs. displacement curves for 20° angle and all types of mortar.

Taking into account the results of the strength tests and economic considerations, a brittle fracture toughness test was carried out for samples containing 2% fibres. In order to assess the impact of the presence of fibres on the ductility and stiffness of epoxy mortars, Figure 14, Figure 15 and Figure 16 contain typical load curves as a displacement function for mortars modified with three types of fibres and control samples, for force angles 0°, 10° and 20° respectively.

It can be seen in Figure 14, Figure 15 and Figure 16 that the curves obtained have a very similar shape before reaching the peak load. After peak load, unstable crack propagation occurs. After this point, it can be seen that samples reinforced with glass fibres show post-peak resistance probably due to composite mode failures still in progress, but failure has already occurred. Similar conclusions for epoxy mortars with the addition of glass fibres were noted by the authors of [16]. This behaviour is particularly evident in mortars containing carbon fibres for 0° and 10° angles. At an angle of 20°, the curve is slightly different after the peak load, but the destruction for samples with carbon and glass fibres occurred at a much higher force than that for controls and with polypropylene fibres. The highest breaking load for each type of fibre occurred for the crack angle of 0°.

**Figure 17:**
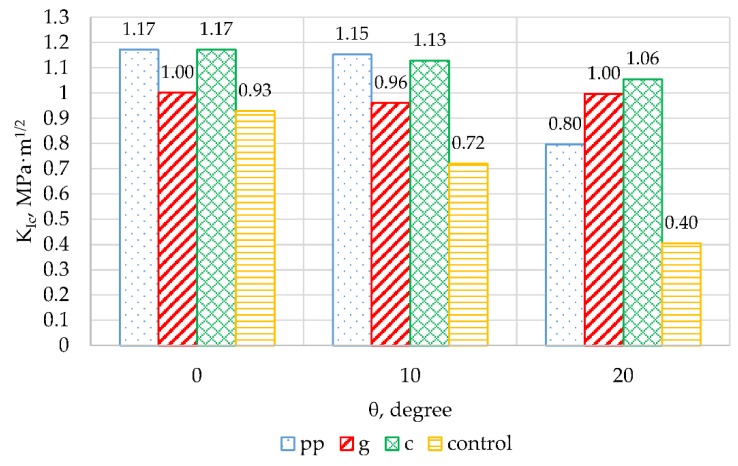
K_IC_ for all types of mortars for different crack inclination angles.

**Figure 18:**
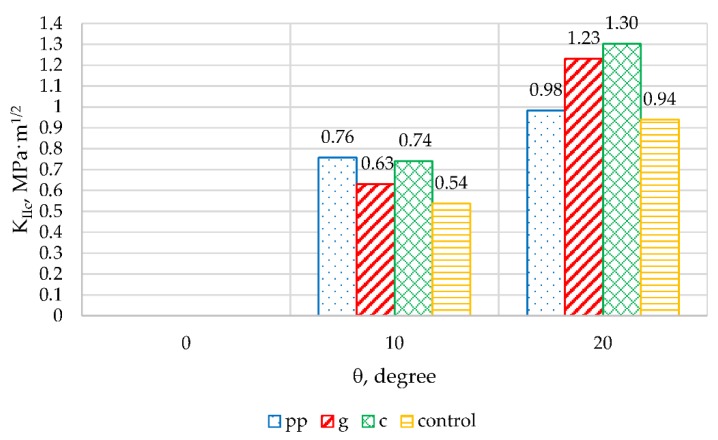
K_IIC_ for all types of mortars for different crack inclination angles.

Figure 17 and Figure 18 summarize, respectively, the K_IC_ and K_IIC_ coefficients calculated for control samples and those with fibres, for various angles of application of force in relation to the arrangement of the metal plate.

Taking into account the first cracking model and the values of the obtained K_IC_ coefficients (Figure 17), it can be noted that the addition of fibres causes an increase in the K_IC_ coefficient, and thus improves the brittle fracture toughness of epoxy mortars. For the angles 0° and 10° the values of this coefficient for mortars containing polypropylene and carbon fibres are very similar. When changing the angle to 20°, K_IC_ for samples containing carbon fibre the value of this coefficient was only slightly reduced by 6.2%, but for those with polypropylene fibres a decrease of over 30% was noted, compared to the results obtained for the angle 10°. The K_IC_ coefficient for mortars with glass fibres for all angles remains at a very similar level and its values are in the range from 1MPa·m^1/2^ to 0.96 MPa·m^1/2^. 

The K_IIC_ factor is similar to K_IC_ for an angle of 10°. The highest value of 0.76 MPa·m^1/2^ was recorded for mortars containing polypropylene fibres. A slightly lower value of this parameter (0.74 MPa·m^1/2^) was obtained using carbon fibres. For mortars containing glass fibres and control samples, the K_IIC_ parameters were 0.63 MPa·m^1/2^ and 0.54 MPa·m^1/2^ respectively. When changing the angle to 20° this factor is higher than at 10°, this time the highest value was found for mortars modified with carbon fibres (K_IIC_ = 1.30 MPa·m^1/2^).

The obtained coefficients have higher values if we compare them with the results obtained in [16]. This may be due to the fact that the fibres were 12 mm long, i.e. they were twice as long as those used in the research conducted by Moreira et al. [16]. As demonstrated by the authors of [15], the adhesion between fibre and matrix strongly depends on the orientation of particularly short fibres. There are differences in the distribution of stresses along the fibre-matrix border in the case of short fibres.

### 3.4. Scanning Electron Microscopy (SEM)

Through the images obtained by SEM, it was possible to verify the incorporation in the resin, the dispersion in the matrix and the behaviour of the fibre after the application of the tensile and compression loads.

**Figure 19:**
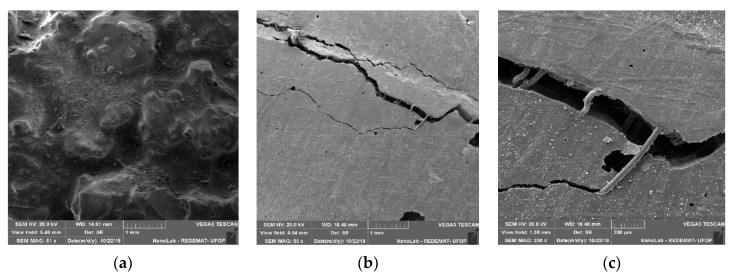
Flexural (**a**) (51× magnification) and lateral (**b**) (50× magnification), (**c**) (200× magnification) surface of the matrix of epoxy resin and glass fibre.

In the matrix of epoxy resin and glass fibre, there was uniform dispersion of the fibres, in addition, it is observed that the fibres were well incorporated in the resin. The fracture surface had a wavy behaviour, indicating that plastic deformation occurred during load insertion [13] (Figure 19a). As shown in Figure 19b,c it can be observed that the glass fibres acted as a matrix skeleton, supporting the load applied to the composite. When applying the loads, the fibres can absorb and resist part of the load, preventing the matrix from being damaged.

**Figure 20:**
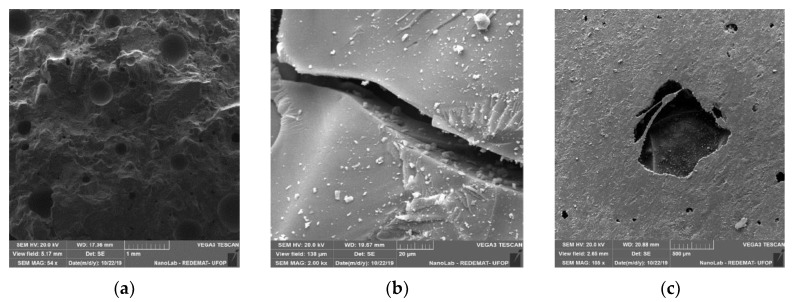
Flexural (**a**) (54× magnification), (**b**) (2000× magnification) and lateral (**c**) (105× magnification) surface of the matrix of epoxy resin and carbon fibre.

Figure 20a,b show that the samples with carbon fibre, in the fracture regions, fibres oriented perpendicular to the rupture were not identified. Figure 20c presents a carbon fibre within the pore of the epoxy matrix. With this analysis, carbon fibre was not as easily evident compared to polypropylene and glass fibre resins.

**Figure 21:**
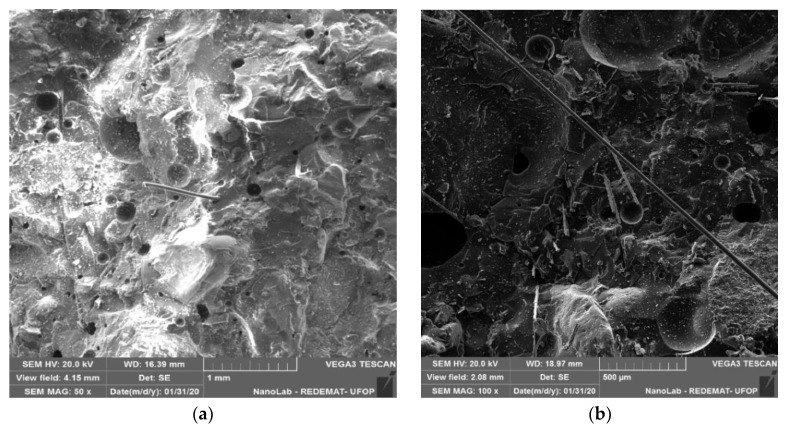

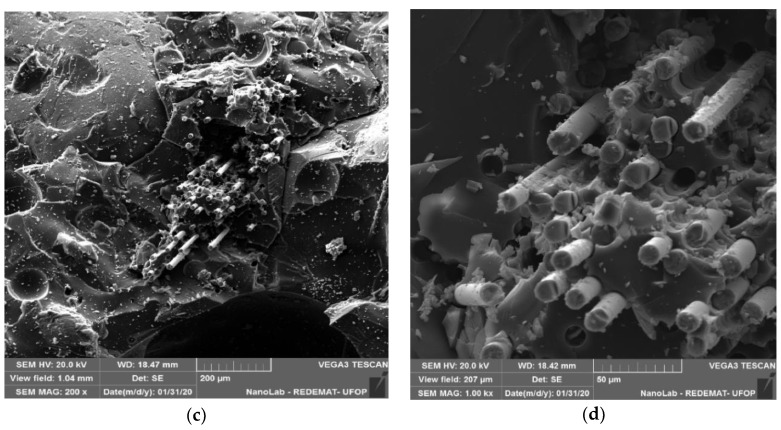
Flexural surface of the matrix of epoxy resin and polypropylene fibre (**a**) 50×; (**b**) 100×; (**c**) 200×; (**d**) 1000× magnification.

Figure 21a shows the SEM images of fracture surface of samples of mortars containing polypropylene fibres had a homogeneous dispersion in the matrix. Figure 21b shows a part of the fractured surface of the specimen with 5% polypropylene fibres, where there is a concentration of fibres in the direction perpendicular to the break. Figure 21c,d are close-ups of 21b, indicating a break in the adhesion between epoxy resin and polypropylene fibre. The holes in Figure 21d suggest that the fibres were pulled out from the other part of the specimen instead of breaking the fibre. This corroborates the drop in mechanical strength from 4% to 5% fibre replacement.

**Figure 22:**
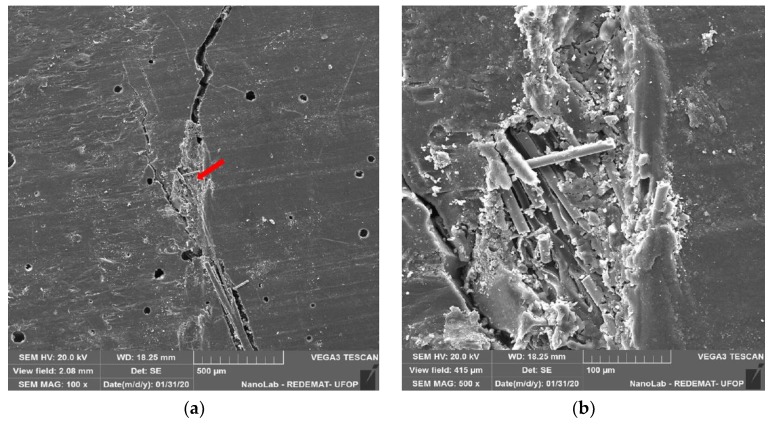
Flexural surface of the matrix of epoxy resin and polypropylene fibre (**a**) 100×; (**b**) 500× magnification.

In Figure 22, in the matrix of epoxy resin with polypropylene fibre, it is possible to observe non-dispersed fibres oriented towards the crack. With the accumulation and orientation of the fibres, a fragile region appears, where the crack propagation occurs.

## 4. Conclusions

For this article, tests of epoxy mortars with the addition of three types of fibres were performed to assess the effect of modifications on the mechanical properties of mortars and their brittle fracture toughness. The following conclusions can be drawn:

- Addition of each of the three types of fibre in an amount of up to 4% wt. allows the obtaining of mortars with higher flexural and compressive strength compared to control mortars. The highest values of flexural strength (25.68 MPa) were obtained at 2% substitution of sand with polypropylene fibres. The most favourable compressive strength, equal to 100.98 MPa, was also noted for mortars with polypropylene fibres (addition at the level of 4%).
- Replacing sand 2% by weight with one of three types of fibres, respectively, increases the stress intensity factors and, compared to the control mortars, improves crack resistance.
- Analysing the graphs depicting the force-displacement relationship, it can be observed that at the time of breaking, cracking in mortars reinforced with polypropylene fibres spreads faster than with mortars containing glass and carbon fibres. The SEM analysis confirmed that in the matrix of epoxy resin with polypropylene fibre, it is possible to observe non-dispersed fibres oriented towards the crack. With the accumulation and orientation of the fibres, a fragile region appears, where the crack propagation occurs.
- Based on the obtained research results, and also due to availability and price, the most favourable seems to be the production of composites containing the addition of polypropylene fibres.

## Figures and Tables

**Table 1:** The physico-chemical properties of Epidian 5 resin [29].

Type of Resin	Density, g/cm^3^	Viscosity 25 °C, MPa∙s	Molecular Weight, g/mol	Epoxy Count LE, mol/100 g
Epidian 5	1.17	30,000	450	0.49

**Table 2:** Selected physico-chemical properties of curing agent used in the tests [29].

Type of Curing Agent	Density 20 °C, g/cm^3^	Viscosity 25 °C, MPa∙s	Amine Number, mg KOH/g	Form	Main Ingredient
Z-1	0.978–0.983	20–30	min. 1100	Liquid pale yellow	triethylenetetramine

**Table 3:** Selected properties of fibres used in the tests.

Type of Fibre	Designation	Length, mm	Density, g/cm^3^	Cost
Polypropylene	pp	12	0.91	Lowest
Glass	g	12	2.50	Intermediate
Carbon	c	12	1.60	Highest

**Table 4:** List of average values of mortar flexural strength with standard deviation.

Fibre Content %	Flexural Strength, MPa		
	PP Fibres	Glass Fibres	Carbon Fibres
0	22.55 ± 0.53	22.55 ± 0.53	22.55 ± 0.53
1	23.03 ± 1.96	23.49 ± 1.00	22.77 ± 0.75
2	25.68 ± 1.57	24.60 ± 0.43	22.87 ± 1.31
3	25.15 ± 0.09	24.48 ± 1.66	23.58 ± 1.14
4	24.16 ± 2.87	23.33 ± 1.13	22.78 ± 0.15
5	23.84 ± 0.52	21.13 ± 0.92	22.55 ± 1.00
Total	24.07 ± 1.75	23.26 ± 1.49	22.85 ± 0.84

**Table 5:** Results of the Kruskal-Wallis ANOVA rank test for flexural strength related to the type of fibres.

Dependent Variable: Flexural Strength, MPa	ANOVA Rank Kruskal-Wallis; Flexural Strength, MPa Independent (Grouping) Variable: Type of Fibre Kruskal-Wallis Test: H (2, N = 54) = 5.398859; p = 0.0672		
	N Important	Total Ranks	Average Rank
pp	18	607.50	33.75
g	18	489.00	27.17
c	18	388.50	21.58

**Table 6:** Values p for two-sided comparisons.

Dependent Variable: Flexural Strength, MPa	Value p for Multiple (Two-Sided) Comparisons; Flexural Strength, MPa Independent (Grouping) Variable: Type of Fibre Kruskal-Wallis Test: H (2, N = 54) = 5.398859; p = 0.0672		
	pp R:33.75	g R:27.17	c R:21:58
pp		0.628009031	0.0610076543
g	0.628009031		0.861035278
c	0.0610076543	0.861035278	

**Table 7:** Results of the Kruskal-Wallis ANOVA rank test for flexural strength related to fibre content.

Dependent Variable: Flexural Strength, MPa	ANOVA Rank Kruskal-Wallis; Flexural Strength, MPa Independent (Grouping) Variable: Amount of Fibre, % Kruskal-Wallis Test: H (5, N = 54) = 15.00301; p = 0.0104		
	N Important	Total Ranks	Average Rank
0	9	160.50	17.83
1	9	213.00	23.67
2	9	339.00	37.67
3	9	351.00	39.00
4	9	248.50	27.61
5	9	173.00	19.22

**Table 8:** Values p for two-sided comparisons.

Dependent Variable: Flexural Strength, MPa	Value p for Multiple (Two-Sided) Comparisons; Flexural Strength, MPa Independent (Grouping) Variable: Amount of Fibre, % Kruskal-Wallis Test: H (5, N = 54) = 15.00301; p = 0.0104					
	0 R:17.83	1 R:23.67	2 R:37.67	3 R:39.00	4 R:27.61	5 R:19.22
0		1.000000	0.112319	0.064736	1.000000	1.000000
1	1.000000		0.885873	0.580241	1.000000	1.000000
2	0.112319	0.885873		1.000000	1.000000	0.193212
3	0.064736	0.580241	1.000000		1.000000	0.114854
4	1.000000	1.000000	1.000000	1.000000		1.000000
5	1.000000	1.000000	0.193212	0.114854	1.000000	

**Table 9:** List of average values of mortar compressive strength with standard deviation.

Fibre Content, %	Compressive Strength, MPa		
	PP Fibres	Glass Fibres	Carbon Fibres
0	93.85 ± 2.55	93.85 ± 2.55	93.85 ± 2.55
1	94.37 ± 2.64	98.87 ± 2.53	96.87 ± 2.36
2	100.00 ± 1.55	99.55 ± 1.46	98.13 ± 1.81
3	100.38 ± 1.02	100.03 ± 2.66	97.72 ± 2.07
4	100.98 ± 1.40	99.10 ± 3.22	96.20 ± 1.11
5	96.07 ± 2.01	97.62 ± 3.26	93.03 ± 2.98
Total	97.61 ± 3.49	98.17 ± 3.25	95.97 ± 2.82

**Table 10:** Results of the Kruskal-Wallis ANOVA rank test of compressive strength related to the type of fibres.

Dependent Variable: Compressive Strength, MPa	ANOVA Rank Kruskal-Wallis; Compressive Strength, MPa Independent (Grouping) Variable: Type of Fibre Kruskal-Wallis Test: H (2, N = 108) = 9.134187 p = 0.0104		
	N Important	Total Ranks	Average Rank
pp	36	2112.50	58.68
g	36	2266.50	62.96
c	36	1507.00	41.86

**Table 11:** Values p for two-sided comparisons.

Dependent Variable: Compressive Strength, MPa	Value p for Multiple (Two-Sided) Comparisons; Compressive Strength, MPa Independent (Grouping) Variable: Type of Fibre Kruskal-Wallis Test: H (2, N = 108) = 9.134187 p = 0.0104		
	pp R:58.68	g R:62.96	c R:41.86
pp		1.000000	0.068124
g	1.000000		0.012799
c	0.068124	0.012799	

**Table 12:** Results of the Kruskal-Wallis ANOVA rank test of compressive strength related to fibre content.

Dependent Variable: Compressive Strength, MPa	ANOVA Rank Kruskal-Wallis; Compressive Strength, MPa Independent (Grouping) Variable: Amount of Fibre, % Kruskal-Wallis Test: H (5, N = 108) = 44.22612; p = 0.0000		
	N Important	Total Ranks	Average Rank
0	18	400.50	22.25
1	18	869.00	48.28
2	18	1344.50	74.69
3	18	1371.50	76.19
4	18	1219.00	67.72
5	18	681.50	37.86

**Table 13:** Values p for two-sided comparisons.

Dependent Variable: Compressive Strength, MPa	Value p for Multiple (Two-Sided) Comparisons; Compressive Strength, MPa Independent (Grouping) Variable: Amount of Fibre, % Kruskal-Wallis Test: H (5, N = 108) = 44.22612; p = 0.0000					
	0 R:22.250	1 R:48.278	2 R:74.694	3 R:76.194	4 R:67.722	5 R:37.861
0		0.189998	0.000008	0.000004	0.000199	1.000000
1	0.189998		0.170968	0.112452	0.938116	1.000000
2	0.000008	0.170968		1.000000	1.000000	0.006281
3	0.000004	0.112452	1.000000		1.000000	0.003615
4	0.000199	0.938116	1.000000	1.000000		0.063511
5	1.000000	1.000000	0.006281	0.003615	0.063511	

## References

[1] Ribeiro M.C.S., Meixedo J.P., Fiúza A., Dinis M.L., Meira Castro A.C., Silva F.J.G., Costa C., Ferreira F., Alvim M.R. (2011). Mechanical Behaviour Analysis of Polyester Polymer Mortars Modified with Recycled GFRP Waste Materials. World Acad. Sci. Eng. Technol..

[2] Al-Maadeed M.A., Labidi S. (2014). Recycled polymers in natural fibre-reinforced polymer composites, Natural Fibre Composites. Mater. Process Appl..

[3] Dębska B., Dębska B.J., Lichołai L. (2019). Evaluation of the Utility of Using Classification Algorithms when Designing New Polymer Composites. J. Ecol. Eng..

[4] Czarnecki L. (2010). Polymer concrete. Cem. Lime Concr..

[5] Dębska B., Lichołai L., Krasoń J. (2017). Selected properties of epoxy mortars with perlite aggregate. J. Ecol. Eng..

[6] Dębska B. (2018). The use of discriminant analysis methods for diagnosis of the causes of differences in the properties of resin mortar containing various fillers. E3S Web Conferences.

[7] Dębska B., Lichołai L., Miąsik P. (2019). Assessment of the Applicability of Sustainable Epoxy Composites Containing Waste Rubber Aggregates in Buildings. Buildings.

[8] Correia L., Partala T., Loch F.C., Segadães A.M. (2010). Factorial design used to model the compressive strength of mortars containing recycled rubber. Compos. Struct..

[9] Dębska B., Lichołai L. (2018). Long-Term Chemical Resistance of Ecological Epoxy Polymer Composites. J. Ecol. Eng..

[10] Tavares C.M.L., Ribeiro M.C.S., Ferreira A.J.M., Guedes R.M. (2002). Creep behaviour of FRP-reinforced polymer concrete. Compos. Struct..

[11] Ribeiro M.C.S., Nóvoa P.R., Ferreira A.J.M., Marques A.T. (2004). Flexural performance of polyester and epoxy polymer mortars under severe thermal conditions. Cem. Concr. Compos..

[12] Nunes L.C.S., Reis J.M.L., Mattos H.S.C. (2011). Parameters identification of polymer concrete using a fracture mechanics test method and full-field measurements. Eng. Fract. Mech..

[13] Chen P., Wang Y., Li J., Wang H., Zhang L. (2018). Adhesion and erosion properties of epoxy resin composite coatings reinforced with fly ash cenospheres and short glass fibers. Prog. Org. Coat..

[14] Nunes L.C.S., Reis J.M.L. (2012). Estimation of crack-tip-opening displacement and crack extension of glass fiber reinforced polymer mortars using digital image correlation method. Mater. Des..

[15] Reis J.M.L., Menezes E.M. (2017). Barley residue reinforced polymer mortars: Fracture mechanics approach. Compos. Struct..

[16] Moreira G.C., Reis J.M.L., Rohan U., Soares C.A.P., da Costa Mattos H.S. (2016). Effect of fiber reinforcement on mixed-mode fracture of polymer mortars. Compos. Struct..

[17] Alshammari B.A., Saba N., Alotaibi M.D., Alotibi M.F., Jawaid M., Alothman O.Y. (2019). Evaluation of Mechanical, Physical, and Morphological Properties of Epoxy Composites Reinforced with Different Date Palm Fillers. Materials.

[18] Reis J.M.L., Motta E.P. (2014). Mechanical behavior of piassava fiber reinforced castor oil polymer mortars. Compos. Struct..

[19] Meira Castro A.C., Ribeiro M.C.S., Santos J., Meixedo J.P., Silva F.J.G., Fiúza A., Dinis M.L., Alvim M.R. (2013). Sustainable waste recycling solution for the glass fibre reinforced polymer composite materials industry. Constr. Build. Mater..

[20] Xie X., Kang X., Jin Y., Cai J. (2018). The Effect of Mechanical Performance on PP Fiber to Polymer Mortar. IOP Conference Series: Earth and Environmental Science.

[21] Reis J.M.L. (2012). Sisal fiber polymer mortar composites: Introductory fracture mechanics approach. Constr. Build. Mater..

[22] Reis J.M.L., Ferreira A.J.M. (2004). Assessment of fracture properties of epoxy polymer concrete reinforced with short carbon and glass fibers. Constr. Build. Mater..

[23] Reis J.M.L., Chianelli-Junior R., Cardoso J.L., Marinho F.J.V. (2011). Effect of recycled PET in the fracture mechanics of polymer mortar. Constr. Build. Mater..

[24] Reis J.M.L. (2006). Fracture and flexural characterization of natural fiber-reinforced polymer concrete. Constr. Build. Mater..

[25] Reis J.M.L., Carneiro E.P. (2013). Effect of piassava lees in the fracture behavior of polymer mortars. Compos. Struct..

[26] Reis J.M.L., Moreira D.C., Nunes L.C.S., Sphaier L.A. (2011). Evaluation of the fracture properties of polymer mortars reinforced with nanoparticles. Compos. Struct..

[27] Verma D., Gope P., Shandilya A., Gupta A., Maheshwari M. (2013). Coir fibre reinforcement and application in polymer composites: A Review. J. Mater. Environ. Sci..

[28] Adeniyi A.G., Onifade D.V., Ighalo J.O., Adeoye A.S. (2019). A review of coir fiber reinforced polymer composites. Compos. Part B Eng..

[29] Dębska B., Lichołai L. (2016). The effect of the type of curing agent on selected properties of epoxy mortar modified with PET glycolisate. Constr. Build. Mater..

[30] PN-EN 196-1: 2016-07 (2016). Cement Testing Methods—Part 1: Determination of Strength.

[31] Silva Da Costa Mattos H., Reis J., Cardoso Moreira G. Effect of TETRA PAK waste on mixed-mode fracture of polymer mortars using Brazilian Disc Test. Proceedings of the ABCM International Congress of Mechanical Engineering COBEM2015.

